# Review of Dr. Hunter's Third Set of Formulas

**Published:** 1853-01

**Authors:** John Allen


### ARTICLE XIV.

MESSRS. EDITORS.—Will you please copy from the News Letter, my Review of Dr. Hunter's third set of formulas, as *claimed by him*, for continuous Artificial Gums.

Respectfully, Yours, J. ALLEN.

### *Review of Dr. Hunter's third set of Formulas.* BY JOHN ALLEN, D. D. S.

AGAIN we have another set of formulas published by Wm. M. Hunter. This is the third set he has given to the profession within the last year, which promises to be of as much utility, without practical demonstration, as either of the others. And what has been his motive? To this inquiry an answer is found in the result which he has intended to produce, which can be seen by looking at the vein of bitter feelings, which have stood out in such bold relief throughout all his effusions upon this subject, which shows that they have emanated from the baser passions of the heart, viz.—Professional jealousy, malice and revenge; instead of philanthropy, generosity or magnanimity. Therefore no practical good can reasonably be expected to result from evil designs. In the first set of formulas that he published, he stated that they were worthless; why then did he publish them, if not for the reason above named? The second set he regards no better than the first, and although no practical good could be derived from them, he seemed to think their publication might serve his malicious purpose. Having failed to realize his expectations thus far, he then sent to Washington and obtained my formulas, and published them also. In doing this he evidently had two objects in view, one of which was to try to annoy me, and the other to appropriate them to his own use—or such portions of them as would serve his purpose—and then claim them as his own, which he has done. In his last formulas, he exhibits the same malicious spirit towards me that characterized his former articles upon this subject, with a few more falsehoods, which we will notice, although we had come to the conclusion to take the advice of an eminent divine, (Dr. B.) who remarked that he once wrote a whole volume against a skunk, and gained nothing by it, but rather got the worst of it. He therefore advises all persons who desire clean skirts, to let skunks alone. But let us first notice a few paragraphs in his last, "issue of receipts." He commences by saying "that he does not know that he shall offer any thing new to a certain class of readers, but feels convinced that the better informed of the practitioners in our profession will find a practical elimination of good from old ideas." As much as to say that he alone had been able to render old ideas practical. In his next paragraph he states, that to Delabarre must be given the credit of having first conceived and executed the union of artificial teeth with an artificial gum and plate.—Vide Fitch's Dental Surgery. This gum, as described by Delabarre, consisted in a kind of inlaid work, which he called callodantes, which were set in porcelain paste, after being ground upon stones into convenient shapes, and fitted together like joiner's work, which were then united by means of a flux. This, the reader will perceive, was as different from mine as blue is from black.

Delabarre's method was not rendered sufficiently practical to be adopted or brought into use by the profession; therefore cannot be brought as a bar to my patent, on the ground of abandonment. In the next paragraph he gives Audibran the credit for granulated body, and then gravely asks, "where is the new principle in the patent now claimed?" Let any one compare the two methods, and he will see that there is no similarity in them. In his first paragraph, he states that he did not expect to give any thing *new*, but in his *sixth*, he states that *he* is the one who has perfected the body as used by Jones, White & Co. in the manufacture of their teeth. And in the next sentence, he says, "it is applicable to ordinary gold plate as used by dentists generally in the form of block work, and is made by him in continuous arches where a full denture is required." He further says he uses it, on an alloy of gold and platina, 20 and 22 carats fine.

Here he conveys a false impression, by holding out the idea that his (my) gum, and Jones, White & Co's tooth body are identically the same. To prove the falsity of this assumption, one has only to fuse or attempt to fuse a little of Jones, White & Co's tooth body upon a gold plate, and then he will see the bait that is thrown out for effect.

He next proceeds to prove (what no one disputes) that platina has long been used for dental purposes, and then strings together three or four falsehoods, (merely to give *point* to his preceding remarks) in the following order: "So it will hardly do to set up the claim for *novelty* at this late date; the *patentee*, however, is excusable, for as *he does not read*, *he presumed* that it was original *in the practice* from which *he pilfered it*, and so *originated it*."

With reference to the use of platina, my remarks in its favor were in reply to his, against the use of it, in which I stated that I preferred it as a base for this style of work. That I claim its introduction for dental purposes is *false;* it cannot be found in my specifications. That I do not *read* is *false.* That I *pilfered it* is *false.* That I *originated it* is *false.* In the next sentence he states, that since he first put work into the mouth, his modes have changed very much. No doubt of it, especially since he sent to Washington and obtained my specifications, for he now uses the same ingredients which are embodied in my formulas, and he now finds that good asbestos is a valuable component, which he did not know until he obtained a knowledge of the fact from me. He also learned from my specifications, that platina scraps, united to the plate of gold, for the body to cling to, together with the cohesive properties of the compound, formed a strong union between the gum, plate and teeth, and he can now see how it is, that teeth mounted in this way can resist such strong force. He next proceeds to compliment Dr. Wildman, the object of which is easy to be seen, and then proceeds to give formulas, which we will pass over without further reference, and merely notice some of the last remarks with reference to me. He thinks my vindication the most childish and contemptible effusion he had ever seen. Be it so. I did not study his taste or interest when I wrote it. I thought it only necessary to notice the main points in which my interests were involved. The first point that I endeavored to establish was, that I had made a valuable improvement in setting teeth; and another point was to show that great efforts had been made to wrest it from me—that in spite of those efforts I was fully able to substantiate my claims. Another was, that my method is original with me, and I am not indebted to any other person for it. These I regarded as the main points worthy of attention. But we will now notice some of the others, although of minor importance. He says that Dr. Brown described to me a piece of *his* work two months before I had entered my caveat, and endeavors to convey the idea, that it was not until after Dr. Brown gave me a description of *his*, that I attempted any thing of the kind myself. This is *false.* Dr. Brown testifies that he saw work of that kind that I had put into the mouth more than a year before, and that he was cognizant of the fact that I had been endeavoring for years to perfect a system by which this could be done, and that I was in the habit of getting materials of him with which to conduct my experiments. Now, will the reader look at the facts with reference to this minute description given me by Dr. Brown, upon which he, Dr. H., lays so much stress; long after I had been producing work of this kind, which fact was well known to Dr. B., he one day remarked to me that Dr. Hunter was getting up something similar; I asked him if the teeth were united to each other and to the plate like mine, and he said he could not tell how it was done, not being a dentist, but he saw a piece that he was going to send to the World's Fair, and that the gum appeared to be continuous and looked like block work, and that was all that passed between us upon the subject. So much for the minute description given me by Dr. Brown. His next sentence reads thus: Even John Allen himself is not so reckless of his reputation as to hazard the assertion that I could not have proved priority, &c. I do assert most positively, that he cannot show work of this kind that he did prior to my having executed it. On the contrary, it was long after I had first produced it that he did any thing of the kind, and it was the fact of my doing it that prompted him to make the efforts he has to effect the same ends, and that he and his clique have done every thing in their power to wrest it from me. The means which have been employed were calumny and falsehood—but the object was to first put me down and then rob me of my invention. In attempting to do this, they found they had a formidable task to perform. Hence the various tactics that have been resorted to, to accomplish that object.

He further asserts that the "Miss. Valley Ass. of Dental Surgeons declared at sight in favor of it," &c. That is false. The society appointed a committee one day, and that committee reported the next, having at least twenty-four hours to test it in acids, try the strength of the material, &c., and then reported the result of their investigation to the meeting; whereupon the society passed a vote of commendation. He then refers to another false charge with reference to Steemer, which I have refuted again and again, with ten or a dozen positive affidavits.

He thinks me very impudent or very foolish to call upon the unfledged noviciates of the Ohio school and sixty dentists to controvert his assertions. Such an array of testimony does not suit his fancy. Again, he says, "failing in my first attempt at imitating what *he* had accomplished, I again tried my imitative powers, and now claim as a part of my patent the soldering of teeth to the plate." It is false that I attempted to imitate what he had accomplished. I never saw any thing of the kind that he ever did until long after I had been doing this style of work. On the contrary, he called at my house to see some of my new style of work. I showed him several specimens, and he then remarked that he was going to make some, and when he got them done he would show them to me, and in some few weeks after that he brought and showed me a piece that he had made, which was the first I had seen of his make. The *imitation* was got up by *him*, not by me. And I do again assert that the first continuous gums he ever saw fused upon the teeth and plate were done by *me*, and that several years ago, I embodied this principle and applied it practically for my patients. Again, he says, "No man in his common senses would ever dream of soldering the teeth to the plate." Now, if he will look again at my letters patent he will find the following language:

"This compound should be intermixed or underlaid with gold and platina scraps. These form a metallic union with the plate upon which the teeth are set." And if he will look a little further he will find the following:—"Although back plates may be attached to the teeth if desired." Either of these methods may be adopted without conflicting with the spirit of my patent. Therefore his charge against me of fraud, is also *false*.

Again, he says, I "also attempt to show that his work is merely block work soldered on." We have his own words for it, as published in the Dental Recorder, and also a still more clear description of which is given in the fourth number of the fifth volume of the Dental News Letter, which states that "Dr. Hunter's *claimed* improvement consists in uniting single teeth together and then mounting them by soldering to the plate." Therefore it is *he* that attempts to mislead the reader, not me.

He next states "that one of the specimens described to me was on platina plate." I did not know it. Only one piece was spoken of to me by Dr. Brown, and so indefinite was his description that he did not state what plate it was on. In the next sentence he asserts, that "I only got platina plate to experiment with, on learning that he was using it, (this is false,) he not having taken all that was ordered for him." I never knew he ordered any. But again, he thinks it the height of impudence for me to use platina base, because Dr. Leslie had *claimed* it for *him*. Of course then it was very impudent in me to say that I preferred it as a base when Dr. H. wanted the credit of it. He next repeats the inquiry, "should a fracture occur, what are the means of repair that would prevent a recurrence of the evil?" In answer to this inquiry I would state that it is much more easily repaired than ordinary block work, for we do not have to carve and bake a new set of teeth and blocks and then mount them as at first, but simply grind off the gum to the plate, and if it is too weak, strengthen it with additional metal, and then put on a new gum and fuse it, and the work will be perfect again, with the same set of teeth, without the trouble of taking the teeth off the plate and then remounting them again.

His next sentence reads thus, "Now for a few more of the main points that he has not thought it necessary to answer." I thought them irrelevant and unworthy of notice, being of the same stamp, emanating from the same source, and fraught with the same evil spirit which has marked his whole course towards me.

"I accused him of an attempt to bribe."

This accusation is false, and is based upon the following trivial circumstance. In speaking with Mr. Tomin with reference to employing Steemer, he said Steemer owed him borrowed money, and he wished he could get into some business that would enable him to pay it back. I remarked that if he worked for me, a portion of his wages might perhaps be applied to paying it off. So much for this charge. Again, he says—

"I accused him of surreptitiously obtaining a patent with a fraud upon the face of it, and he has not answered it."

In reply to this charge I do assert most positively that it is *false*, and there is not a shadow of truth in it. Again—

"I accused him of prevarication, and disgraceful evasion, before the American Society, and he has not answered it."

I doubt whether the American Society has ever called upon Dr. Hunter to demand of me redress for grievances which it has not felt or known as coming from me. But he seems to think himself attorney general for all the Dental Societies, although not a member of any, nor was he present at either of the meetings referred to, and proceeds to bring another charge as follows—

"I accused him of having offered to pay for a gold medal to be awarded by a society, and he has not answered it."

I never made such an offer to a society, and I appeal to the society for the truth of my assertion. But in the course of a conversation with Dr. Leslie at the Louisville Hotel, during recess for dinner, he (Leslie) expressed himself as being opposed to making the award of a medal, which had been proposed before the society. After assigning his reasons, which were various, I remarked to him that I did not wish the society taxed on my account, and that I would not accept of any funds of the society; that the award stamped the value and not the few dollars that it might cost, knowing, as I did full well, that the amount was no consideration with the society; that was not a point which could have any weight with the association. But Dr. Leslie being Dr. Hunter's right-hand man, and having become an inveterate enemy of mine, has made all the capital out of it that he could, and Dr. Hunter retails it out in order to excite public prejudice against me. Again he says—

"I accused him of wilful fraud in placing Brown's note in such juxtaposition, and he has not answered it."

Juxtaposition to what? I do not know what he means. I sent a note to Dr. Brown, and he returned the following answer:

*Cincinnati*, Nov. 18th, 1851.

Dear Doctor.—I have received your note, making inquiries in reference to your improved method of mounting mineral teeth upon metallic plates, and asking how long since I first saw yours worn in the mouth. You called my attention to it in the mouth of a gentleman, then living in this city, two years ago the coming winter. Respectfully, yours,

J. M. BROWN.

Dr. J. ALLEN.

Now, where the wrong is in thus eliciting a simple fact, I do not know. I asked him a plain question and he gave me a candid answer, stating the *facts* as he knew them; but such facts do not suit the doctor's taste. "Unwelcome truths he knows not how to bear."

His next accusation reads thus:—

"I accused him of making a false claim, when he claimed to have overcome shrinkage, and he has not answered it."

I now state that I can place my compound, when properly prepared, upon a full arch of teeth, and after it is fused upon the teeth and plate it will be found that the teeth have not changed their position in the least, neither is the plate drawn out of place in consequence of undue contraction, and the gum will be continuous without crack or blemish. His next charge, I have answered again and again, and therefore deem it unworthy of further notice. Finally, his last accusation reads thus—

"I accused him of being an *ass*, and he *has* answered it."

In reply to this, I would merely state, that I ever regard low blackguardism as unworthy of notice, and therefore always treat it with silent contempt, therefore I have *not* answered it.

His little story that he next introduces, may pass without comment.

In his concluding sentence he bristles up, and snaps and snarls away as saucy as Trip, and says, "he will furnish the testimony if I will enter suit against him," &c. I prefer furnishing the evidence myself, for it is not customary for an *offender* to furnish the documents with which to convict himself.

As the question of priority is one of the points to be kept in view, I will here append a certificate from a gentleman I happened to meet with a short time since in this city, (New York,) who is now wearing a set of teeth put up upon my improved method three years ago.

"I hereby certify, that in the year 1848 I visited Cincinnati, Ohio, and being acquainted with Doctor Allen's family, was a frequent visitor at his house. It was during my first visits that I was shown by the Doctor some specimens of artificial teeth, with gums flowed in upon the plate and teeth, which appeared very beautiful.

"Dr. Allen informed me that he had been several years in preparing this gum. I saw him frequently in his laboratory, working at it, and often until a late hour of the night, trying further experiments in order to bring it to still greater perfection if possible. At a subsequent period, being in the winter of 1849 and '50, Doctor Allen executed a piece of work for me, upon which he flowed his gum, and although they have been in constant use ever since, yet the gum remains perfectly good, which I consider the ultimatum of dentistry.

"I showed it at the time to several persons, who admired it very much, for the very natural appearance of the gum and the beauty of the workmanship. G. MINER HATCH.

"New York, Oct. 20, 1852.

"Sworn to before me, this 26th day of October, 1852.

"H. K. FROST, *Com. of Deeds.*"

Also the following:—

"I hereby certify, that while acting in the capacity of agent for Morris Levett's patent enamel, I called upon John Allen, of Cincinnati, Ohio, on or about the 24th day of October, A. D. 1849; and in conversation with Mr. A. upon the subject of enamels, saw some specimens which Mr. A said he had done; also that he had been experimenting for some time previous to our interview.

"Given under my hand and seal, this 16th day of October, A. D. 1852 THEODORE F. ENGLEBRECHT."

These are only two among many that I shall introduce at the proper time. J. ALLEN.

